# Suppressing FXR promotes antiviral effects of bile acids *via* enhancing the interferon transcription

**DOI:** 10.1016/j.apsb.2024.05.005

**Published:** 2024-05-13

**Authors:** Xue Liang, Kunpeng Liu, Xin Jia, Cuiqin Cheng, Meiqi Zhang, Lingdong Kong, Qiqi Li, Zhe Liu, Min Li, Junliang Li, Yao Wang, Anlong Xu

**Affiliations:** a School of Life Sciences, Beijing University of Chinese Medicine, Beijing 100029, China; b Guangxi Key Laboratory of Special Biomedicine; School of Medicine, Guangxi University, Nanning 530004, China; c Beijing Academy of Traditional Chinese Medicine, Beijing University of Chinese Medicine, Beijing 100029, China; d Beijing Research Institute of Chinese Medicine, Beijing University of Chinese Medicine, Beijing 100029, China

**Keywords:** Bile acids, FXR, IRF3, Interferon transcription, RNA virus, *Z*-guggulsterone, Broad-spectrum antiviral activity, Synergistic inhibitory effect

## Abstract

Bile acids (BAs) are natural metabolites in mammals and have the potential to function as drugs against viral infection. However, the limited understanding of chenodeoxycholic acid (CDCA) receptors and downstream signaling, along with its lower suppression efficiency in inhibiting virus infection limits its clinical application. In this study, we demonstrate that farnesoid X receptor (FXR), the receptor of CDCA, negatively regulates interferon signaling, thereby contributing to the reduced effectiveness of CDCA against virus replication. FXR deficiency or pharmacological inhibition enhances interferon signaling activation to suppress virus infection. Mechanistically, FXR impairs the DNA binding and transcriptional abilities of activated interferon regulatory factor 3 (IRF3) through interaction. Reduced IRF3 transcriptional activity by FXR–IRF3 interaction significantly undermines the expression of Interferon Beta 1 (IFNB1) and the antiviral response of cells, especially upon the CDCA treatment. In *FXR*-deficient cells, or when combined with *Z*-guggulsterone (GUGG) treatment, CDCA exhibits a more potent ability to restrict virus infection. Thus, these findings suggest that FXR serves as a limiting factor for CDCA in inhibiting virus replication, which can be attributed to the “signaling-brake” roles of FXR in interferon signaling. Targeting FXR inhibition represents a promising pharmaceutical strategy for the clinical application of BAs metabolites as antiviral drugs.

## Graphical abstract

FXR inhibition enhances antiviral potency of chenodeoxycholic acid by relieving FXR interference with interferon signaling, indicating FXR as a promising target for augmenting bile acids’ antiviral effects.

**Figure:**
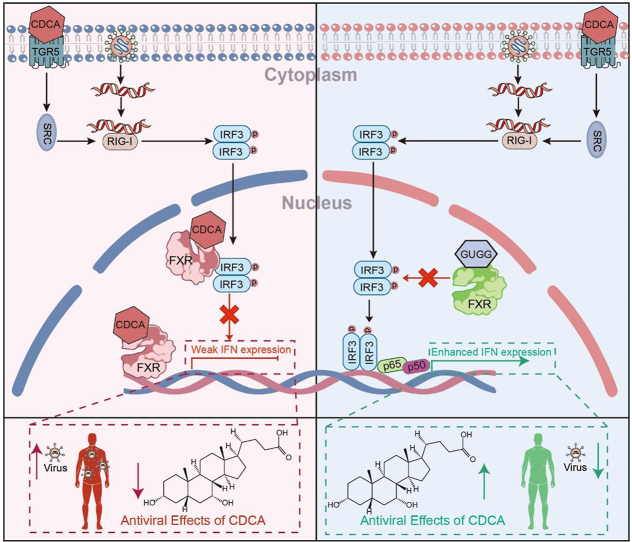

## 1 Introduction

As typical metabolites of mammalian cholesterol metabolism, bile acids (BAs) are synthesized in the liver from cholesterol to form bile, along with cholesterol, phospholipids, and bilirubin^1^. Primary bile acids, such as cholic acid (CA) and chenodeoxycholic acid (CDCA), and secondary bile acids, such as deoxycholic acid (DCA) and lithocholic acid (LCA), are the main types of BAs. Due to their amphipathic nature, BAs are regarded as crucial detergents that facilitate the absorption and transport of dietary lipids, nutrients, and vitamins in the intestines^2^. Accumulating evidence suggests that BAs function as versatile signaling regulators by targeting specific receptors. G protein-coupled receptors (GPCRs) positioned on the cellular membrane such as Takeda G-protein receptor 5 (TGR5)^3^, and nuclear hormone receptors including farnesoid X receptor (FXR)^1^, pregnane X receptor (PXR)^4^, constitutive androstane receptor (CAR)^5^, and vitamin D receptor (VDR)^6^, are the main receptors for sensing BAs. Through the activation of these receptors and downstream signaling pathways, BAs participate in diverse biological processes, particularly those associated with immune responses, effectively bridging metabolism with immunity. The innate immunity of the intestines against chikungunya virus infection requires the involvement of BAs^7^. Specifically, LCA effectively inhibits the replication of porcine delta-coronavirus *in vitro* by activating the GPCR–interferon–ISG15 signaling pathway^8^. Regarding the regulation of host cell antiviral activity, early studies have shown that CDCA inhibits the transcription of target genes in the interferon signaling pathway^9^. In terms of inflammation, BAs can effectively inhibit the activation of the NLRP3 inflammasome by activating the TGR5–cAMP–PKA signaling pathway^10^. Obeticholic acid (INT-747), an FXR agonist, can improve ileal barrier function by reducing intestinal inflammation^11^. Therefore, BAs can directly influence the immune responses of cells.

The immune function of BAs hinges on signaling pathways mediated by BAs and their respective receptors, notably TGR5 and FXR. BAs sensed by TGR5 are involved in the innate immune response of cells^12^. Silencing and antagonizing FXR using *Z*-guggulsterone (GUGG) blocked HCV replication, indicating an inhibitory role of FXR in the antiviral response of hepatic cells^13^. A recent study demonstrated that ursodeoxycholic acid (UDCA), an FDA-approved drug for treating primary biliary cholangitis, can downregulate the expression of ACE2 and prevent SARS-CoV-2 invasion by impeding the co-transcriptional activity of FXR^14^^,^^15^. This finding suggests a potential role for BA receptors in RNA viral infection and interferon signaling. However, evidence of the specific role and detailed mechanism of FXR in RNA viral infection is limited.

Recent studies have shown that the BAs receptor TGR5 promotes type I interferon production through the AKT/IRF3 signaling pathway during virus infection^16^. It has been reported that TGR5 can activate the TGR5–*β*-arrestin–SRC axis, leading to elevated tyrosine phosphorylation of multiple antiviral signaling components. Despite TGR5 being recognized as a significant enhancer of antiviral activity of host cells, the antiviral efficacy of CDCA, a potential agonist of TGR5 (EC_50_ = 6.7 μmol/L) and FXR (EC_50_ = 0.29 μmol/L), remains clinically unsatisfactory. It is demonstrated that CDCA has the ability to restrict the intracellular replication of VSV only at high concentrations (>100 μmol/L) through the TGR5–*β*-arrestin–SRC pathway^12^. The divergent effects observed between TGR5 and CDCA in viral inhibition underscores the complex mechanism of action of BAs and their receptors in the host cell's antiviral activity, thereby indicating the involvement of other BAs receptors in interferon signaling transduction.

In this study, we elucidate that FXR functions as a negative regulator of interferon signaling, consequently diminishing the effectiveness of CDCA in curtailing RNA virus infection. The suppressive role of FXR in modulating the interferon signaling pathway is contingent upon its capacity to inhibit the DNA-binding and transcriptional activity of IRF3. Pharmacological inhibition or deficiency of FXR decreases the infection of hepatic cells by various RNA viruses. Therefore, the combined administration of CDCA and an FXR inhibitor exhibits greater potency in augmenting interferon production and inhibiting viral replication compared to CDCA treatment alone. In summary, our study showed that FXR hindered the antiviral effects of CDCA by suppressing the activation of IRF3, thereby disrupting interferon production. This study provides a novel strategy for enhancing the clinical efficacy of CDCA in inhibiting viral infection and broadens the application of CDCA in clinical treatment.

## 2 Materials and methods

### 2.1 Cells and viruses

HEK293T, Huh7 and AML12 cells were purchased from the Cell Bank at the Institute of Biochemistry and Cell Biology, China Academy of Science, and were cultured in Dulbecco's modified Eagle's medium (DMEM; Gibco) supplemented with 10% fetal bovine serum (EXCEL) and 1% GlutaMAX Supplement (Gibco), and maintained at 37 °C under 5% CO_2_. Transient transfections of cells were performed with Lipofectamine 3000 reagent (Invitrogen) according to the manufacturer's instructions. Vesicular stomatitis virus labeled with enhanced green fluorescent protein (VSV-GFP), VSV (Indiana strain), SeV, EMCV, HSV-1, human influenza virus A/Puerto Rico/8/34 (H1N1) (PR8) were stored from our laboratory.

### 2.2 Virus infection *in vivo*

A total of 24 six-week-old C57BL/6 mice were randomly divided into control and experimental groups (6 per group), and cultured at stable room temperature (25 °C) with regular light/dark cycle. For the virus infection *in vivo*, the VSV virus was amplified using Vero cells, and then it was concentrated by sucrose gradient centrifugation. C57BL/6 mice were intraperitoneally infected with VSV (2 × 10^8^ pfu per mouse) and then treated with vehicle or *Z*-guggulsterone (10 mg/kg/day) or CDCA (10 mg/kg/day) by intraperitoneal injection for 5 days. The survival of the infected mice was monitored until the 7th day from virus infection, and analyzed by Kaplan–Meier methods. After sacrifice, the lungs of mice were collected for the analysis of tissue damage. All experimental methods were conducted by the Animal Care and Use Regulations of China, and were approved by the Beijing University of Chinese Medicine Animal Care Committee (protocol No. BUCM-4-2022041303-2016). All methods were reported by ARRIVE (Animal Research: Reporting of in Vivo Experiments) guidelines.

### 2.3 Antibodies and reagents

The primary antibodies used were as follows: monoclonal anti-Flag M2-peroxidase (Sigma), anti-HA-peroxidase (Roche), anti-MYC-horseradish peroxidase (Santa Cruz Biotechnology), monoclonal anti-ACTB/ACTIN beta (Abclonal), anti-GAPDH (Huabio), anti-FXR (Origene), anti-p-TBK1 (Cell Signaling Technology), anti-TBK1 (Cell Signaling Technology), anti-p-IRF3 (Abcam), anti-IRF3 (Huabio), anti-GFP (Huabio). The secondary antibodies used in western blotting were as follows: goat anti-rabbit IgG-HRP (Cell Signaling Technology) and goat anti-rabbit IgG-HRP (Cell Signaling Technology). CF568-fluorescenct goat anti-mouse IgG (H + L; Biotium), Anti-Flag affinity gel was purchased from Bimake. Protein A/G agarose was purchased from Pierce. The chemical reagents used were as follow: CDCA and GW4064 were purchased from Selleck; *Z*-guggulsterone was purchased from Santa Cruz.

### 2.4 Plasmid construction and mutagenesis

The human genes *FXR*, *IRF3* and other interferon signaling components were generated by PCR amplification from a normal liver cDNA library and cloned into Flag-, HA- and MYC-tagged FG-EH-DEST vectors were derived from Cui Jun lab in Sun Yat-sen University. The plasmids of protein mutants were generated using a site-directed mutagenesis kit (SBS Genetech).

### 2.5 Knockout of FXR by the CRISPR-Cas9 system

Construction of the lenti-CRISPR-Cas9 vectors targeting *FXR*/*mIrf3* was performed following a standard protocol. Briefly, gRNA was synthesized annealed and then ligated into the vector at the BsmBI restriction sites. After the package and infection of the Cas9-gRNA vector by using lenti-virus, the single cell clones of infected cells were selected and performed to WB for detecting the efficiency of gene knockout. The gRNA sequences were obtained using an online gRNA design tool as follows:

*GFP*-sgRNA (as control):

Forward: 5′-CACCGGGGCGAGGAGCTGTTCACCG-3′,

reverse: 5′-AAACCGGTGAACAGCTCCTCGCCCC-3′;

Human *FXR*-sgRNA-1:

Forward: 5′-CACCGAAATCTGTGGTTGAACTTGGGG-3′,

reverse: 5′-AAACCCCCAAGTTCAACCACAGATTTC-3′;

Human *FXR*-sgRNA-2:

Forward: 5′-CACCGTTGGAATAATAGGATGACGAGG-3′,

reverse: 5′-AAACCCTCGTCATCCTATTATTCCAAC-3′;

Human *FXR*-sgRNA-3:

Forward: 5′-CACCGCATGGGCGCGTCAGCAGGGAGG-3′,

reverse: 5′-AAACCCTCCCTGCTGACGCGCCCATGC-3′;

Mouse *Irf3*-sgRNA:

Forward 5′-CACCGGGCAAAATCCGCGGTTTCG-3′,

reverse 5′-AAACCGAAACCGCGGATTTTGCCC-3′.

### 2.6 RNA interference

LipoRNAiMAX (Invitrogen) was used for the transfection of siRNAs into cells according to the manufacturer's instructions. The sequences of siRNAs are listed as follows:

Scramble-siRNA: 5′-UUCUCCGAACGUGUCACGUTT-3′;

Human *FXR*-siRNA-1: 5′-CAAGTGACCTCGACAACAA-3′,

Human *FXR*-siRNA-2: 5′-GAGGAUGCCUCAGGAAAUA-3′;

Mouse *Fxr*-siRNA-1: 5′-GCCGUGUACAAGUGUAAGATT-3′,

Mouse *Fxr*-siRNA-2: 5′-CAGGUUUGUUAACUGAAAUTT-3′.

### 2.7 qPCR

Trizol (Invitrogen) was used for extracting total RNA. Prescript RT reagent kit (Vazyme) was reverse transcribed total RNA to cDNA. Real-time qPCR was operated by choosing the Lightcyler 480 Real-Time PCR System (Roche) and SYBR Green PCR Master Mix (Genstar). *hRPL13A*/*mGapdh* was applied to normalization. All primers used in this work are listed in Supporting Information Table S1.

### 2.8 Flow cytometry

Cells were infected with VSV-green fluorescent protein (VSV-GFP) for appropriate time. After PBS wash, the cells were digested with trypsin solution. Then, fresh culture-medium was used to stop digestion. GFP-positive cells were monitored by EasyCyte 6-2L flow cytometer (Guava). All data were treated with Guava Incyte software.

### 2.9 Cytotoxicity assay

3 × 10^4^ cells were seeded in 100 μL cell culture medium in 96-well plates overnight. The next day, a series of dilutions of agents at the appointed concentration was added to the culture medium (100 μL per well). DMSO processing was established as a negative control and baseline. Cell cultures were incubated for 24 h at 37 °C and 5% CO_2_ cell culture incubator. After that, 10 μL per well of CCK8 reagent (Dojindo) was added to cell culture medium, and 96-well plates were incubated for 1 h in a cell culture incubator. The 450 nm absorbance of samples was detected using a multifunctional enzyme marker (Molecular Devices, SpectraMax i3x).

### 2.10 Luciferase assays

HEK293T was transfected with the indicated reporter or controls plasmid together with a firefly luciferase reporter and *Renilla* luciferase reporter. Twenty-four hours later, luciferase activities were measured with the Dual-Luciferase reporter gene assay kit (Beyotime). Data were normalized by calculating the ratio between firefly luciferase activity and *Renilla* luciferase activity.

### 2.11 Immunofluorescence

Firstly, 4% paraformaldehyde was used for cell fixation. Then cells were permeabilized with 100% methanol. Primary antibodies were incubated with cells overnight at 4 °C. After PBS wash, cells were incubated with fluorescent secondary antibodies for 1 h. Hoechst 33342 (Sigma) staining was used to disclose nuclei. Fluorescence images were acquired by confocal a laser scanning microscope (Zeiss LSM880 + Airyscan).

### 2.12 Immunoprecipitation and immunoblot analysis

Cells were washed twice with cold PBS (Boster Biological Technology, PYG0021). The low salt lysis buffer (1.5 mmol/L MgCl_2_ [Sigma], 50 mmol/L HEPES [Sigma], 150 mmol/L NaCl [Sigma], pH 7.4, 1 mmol/L EDTA [Sigma], 1% Triton X-100 [Sigma] including phosphatase inhibitors [Bimake] and protease inhibitors [Bimake]) was used for cell lysis for 0.5 h at 4 °C. For immunoprecipitation, equivalent amounts of protein per group were incubated with anti-Flag antibody-coupled affinity gel or primary antibodies with protein G agarose beads (Pierce) in lysis buffer overnight. After centrifugation for 5 min at 5000×*g*, 4 °C, the beads were washed with lysis buffer. Loading buffer (pH 6.8, 2.5% SDS [Solarbio], 10% glycerol [Sigma], 0.002% bromophenol blue [Sigma], 0.7135 mol/L (5%) *β*-mercaptoethanol [Solarbio], 2.5 mmol/L Tris–HCl [Solarbio]) was applied to resuspend beads, and beads were subjected to SDS-PAGE. After electrophoresis, the protein bands were moved from gel to polyvinylidene fluoride (PVDF) membranes (Millipore). Specific primary antibodies were used to probe the protein bands, following suitable secondary antibodies. ChemiDoc XRS system (Bio-Rad Laboratories, Hercules, CA, USA) worked for inspecting protein bands.

### 2.13 ChIP

Mouse AML12 cells were treated as indicated, and divided into two groups: input group or ChIP group. The input group was performed for RNA extraction and qPCR assay by using of human GAPDH primer (Forward Primer: 5′-TACTAGCGGTTTTACGGGCG-3′; Reverse Primer: 5′-TCGAACAGGAGGAGCAGAGAGCGA-3′). The formaldehyde was added to the ChIP group to a final concentration of 1% for cross-linking of chromatin, which was stopped by 0.125 mol/L glycine for 10 min at room temperature. The chromatin immunoprecipitation assays were performed according to the ChIP assay kit protocol (Beyotime Biotechnology). Briefly, the cells were washed twice with cold PBS containing PMSF and harvested in SDS Lysis buffer from a ChIP assay kit. After sonication of the sample, protein A + G agarose with salmon sperm DNA treatment was used to pre-clear the whole cell lysate for 30 min at 4 °C. The samples were incubated with anti-IRF3 or control IgG antibodies (Beyotime Biotechnology) overnight. Then, protein A + G Agarose with salmon sperm DNA treatment was added for 2 h-incubation at 4 °C. After successive washing with low-salt/high-salt/LiCl immune complex wash buffer and TE buffer (twice) for 5 min at 4 °C rotation, the DNA–protein complexes were eluted with elution buffer (1% SDS and 0.1 mol/L NaHCO_3_) and then de-crosslinked by adding 0.2 mol/L NaCl and heating for 4 h at 65 °C. Then, the proteins were digested with proteinase K for 1 h at 45 °C, and the DNA segments were purified by a DNA Purification Kit (Vazyme) and used for qPCR reaction. The primers for ChIP-PCR are listed:

*mIfnb1*-ChIP-F: 5′-AGGGAGAACTGAAAGTGGGA-3′,

*mIfnb1*-ChIP-R: 5′-GCTACCTGCAAGATGAGGCA-3′.

### 2.14 Statistical analysis

GraphPad Prism 8.0 software was used for all data processing. Quantitative data in histograms express as mean ± standard deviation (SD). Student's *t*-test was used to analyze relevant data. *P* < 0.05 accounts for statistical significance.

## 3 Results

### 3.1 The antiviral efficiency of BAs is impaired by serum supplementation

**Figure 1:**
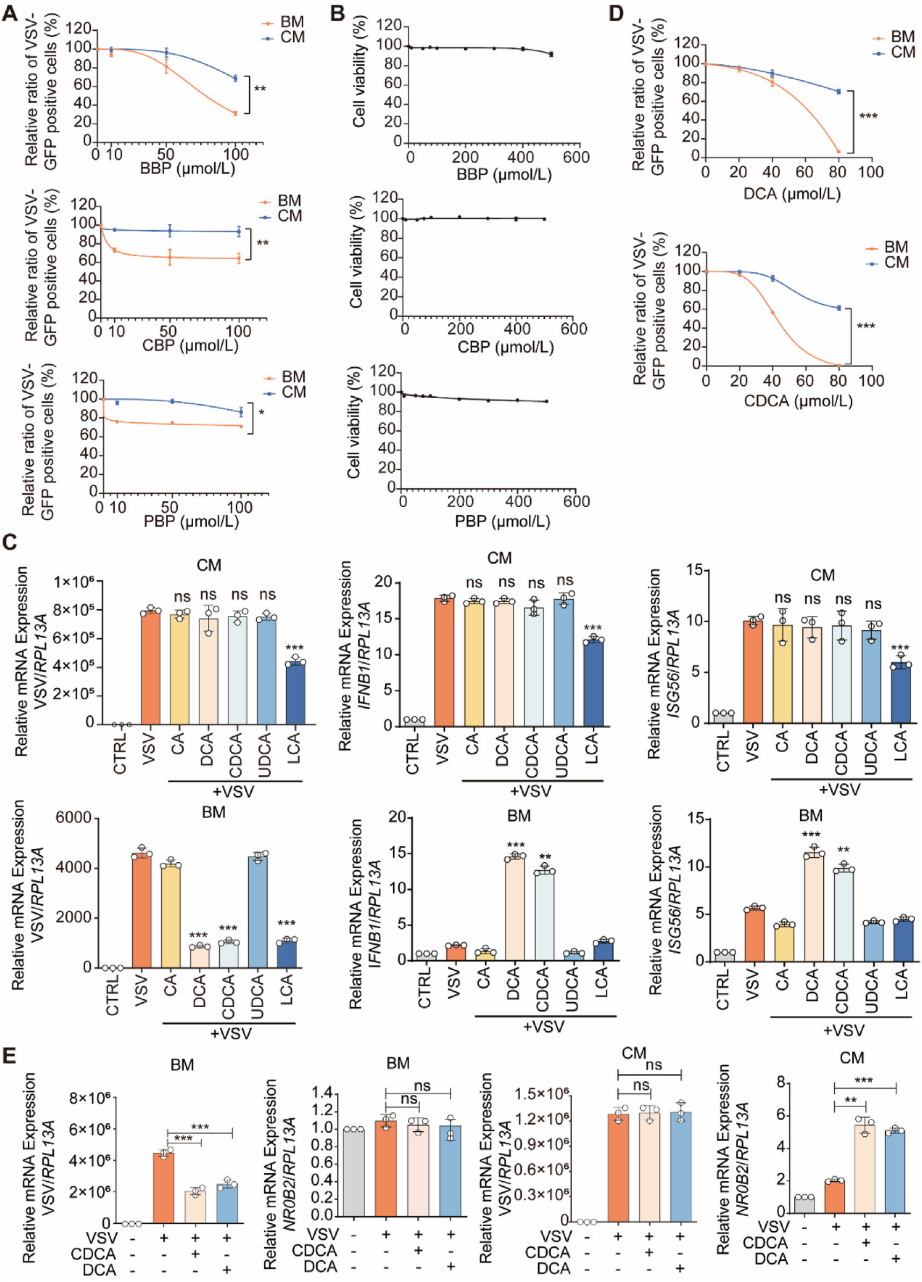
The antiviral efficiency of BAs is impaired by serum supplementation. (A) Flow cytometry analysis of VSV-GFP positive cells ratio in THP1 cells cultured in FBS-containing complete medium (CM) or FBS-free basic medium (BM), and infected with VSV-GFP for 12 h following with or without treatment of BBP (bear bile powder)/PBP (pig bile powder)/CBP (cow bile powder). (B) CCK8 assay analysis of the cytotoxicity of the BBP/PBP/CBP in THP1 cells. Various concentrations of the BBP/PBP/CBP were used to assess their cytotoxicity. (C) qPCR analysis of the VSV, *IFNB1* and *ISG56* mRNA expression in THP1 cells cultured in CM or BM, and infected with VSV for 12 h following with or without treatment of different types of BAs (40 μmol/L). (D) Flow cytometry analysis of VSV-GFP positive cells ratio in THP1 cells cultured in CM or BM, and infected with VSV-GFP for 12 h following with or without treatment DCA/CDCA at various concentrations. (E) qPCR analysis of the VSV and *NR0B2* mRNA expression in THP1 cells cultured in CM or BM, and infected with VSV for 12 h following with or without treatment of DCA (40 μmol/L)/CDCA (40 μmol/L). (A–E) *n* = 3 per group. The data are shown as mean ± SD and are analyzed by Student's *t*-test. ∗*P* < 0.05, ∗∗*P* < 0.01, ∗∗∗*P* < 0.001, ns = no significance.

To mitigate the influence of serum BAs on the antiviral response of host cells, we utilized fetal bovine serum (FBS)-containing complete medium (CM) and FBS-free basic medium (BM) to evaluate the antiviral efficacy of bile powders. The results showed that the three bile powders did not significantly suppress VSV replication under CM condition (Fig. 1A). However, bear bile powder (BBP) exhibited relatively significant activity in inhibiting virus infection at a high concentration (100 μmol/L) in the BM condition (Fig. 1A). The three different types of bile powders exhibited negligible cytotoxicity in THP1 cells (Fig. 1B). This observation demonstrates that the removal of serum can enhance the antiviral potency of BBP. The weak antiviral potency of BBP in CM condition suggests the BAs in FBS impede the efficiency of BBP in inhibiting viruses.

Following, we performed screening experiments to identify the specific BAs responsible for the antiviral properties of BBP in VSV-infected THP1 cells. 40 μmol/L concentration of DCA and CDCA could enhance IFNB1 expression and inhibit virus replication in cells cultured in BM condition, but not in CM condition (Fig. 1C). Moreover, DCA and CDCA inhibited viral replication at 80 μmol/L concentration in cells cultured in CM condition (Fig. 1D). This result is consistent with previous studies in which CDCA can suppress VSV infection in cells at 100 μmol/L concentration *via* activating TGR5, one of the receptors of CDCA^12^. A decrease in the effective antiviral concentration of CDCA (40 μmol/L) was observed in cells cultured in BM medium (Fig. 1D). Thus, the observed differences in the antiviral properties of BBP in cells cultivated in CM/BM could potentially be attributed by the variability in the antiviral effectiveness of CDCA/DCA in CM/BM, indicating that components of the serum-supplemented culture medium may antagonize the antiviral properties of CDCA/DCA. Considering the multiple targets of CDCA and DCA, we studied the activity of the main CDCA/DCA receptor, FXR. As a transcription factor, FXR can induce the expression of target genes such as NR0B2^1^. NR0B2 is a marker of FXR activation. We found that the expression of *NR0B2* was enhanced by DCA/CDCA treatment in THP1 cells cultured in CM, but not in BM (Fig. 1E). Based on this observation, FXR activation may be involved in the limited antiviral efficacy of DCA/CDCA.

### 3.2 FXR is differentially expressed in the hepatic cells or the immune cells

**Figure 2:**
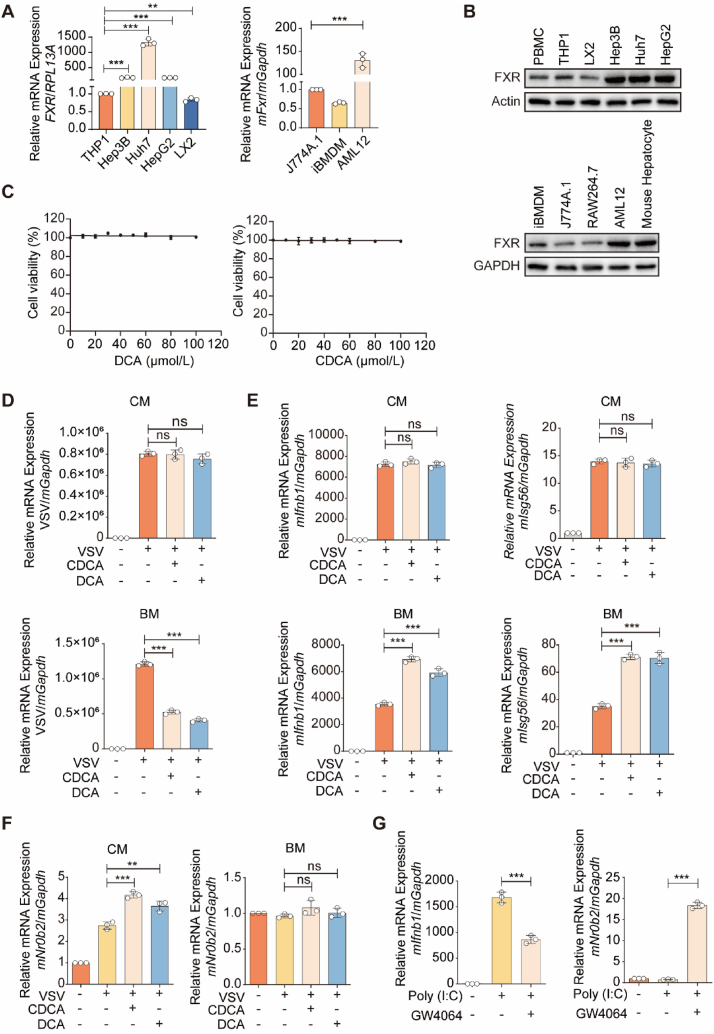
FXR is differentially expressed in the hepatic cells or in the immune cells. (A) qPCR analysis of *FXR* mRNA expression in hepatic cells and immune cells. (B) Immunoblot analysis of FXR level from hepatic cells and immune cells. (C) CCK8 assay analysis of the cytotoxicity of the DCA/CDCA in AML12 cells. Various concentrations of the DCA/CDCA were used to assess their cytotoxicity. (D–F). qPCR analysis of VSV (D), *mIfnb1* and *mIsg56* (E) or *mNr0b2* (F) mRNA expression in AML12 cells cultured in CM or BM, and infected with VSV for 12 h following with or without treatment of DCA/CDCA (40 μmol/L). (G) qPCR analysis of *mIfnb1* and *mNr0b2* mRNA expression in AML12 cells, and then transfected with poly(I:C) for 6 h and treated with or without GW4064 (10 μmol/L). (A) *n* = 3 per group; (C–G) *n* = 3 per group. The data are shown as mean ± SD and were analyzed by Student's *t*-test. ∗*P* < 0.05, ∗∗*P* < 0.01, ∗∗∗*P* < 0.001, ns = no significance.

Since variations in FXR activity of cells in FBS-containing complete medium (CM) and FBS-free basic medium (BM) could potentially influence the antiviral efficiency of CDCA, we investigated the expression of FXR in hepatic cells and immune cells. The mRNA levels of *FXR* were notably higher in human hepatic cells (Hep 3B, Huh7, and HepG2), compared to human immune cells (THP1, LX2). Similarly, in mouse cells, *mFxr* was highly expressed in AML12 cells (mouse hepatic cells) but not in J774A.1 or iBMDM cells (mouse immune cells) (Fig. 2A). Similar results were obtained for FXR protein levels (Fig. 2B). These results suggest that the expression of FXR in hepatocytes is much higher than that in immune cells.

Next, we investigated whether different concentrations of DCA/CDCA were cytotoxic to hepatic cells. The experiments revealed that the DCA/CDCA had no obvious toxicity at 100 μmol/L concentration in AML12 cells (Fig. 2C). Given the safety of DCA/CDCA in hepatic cells, we determined its antiviral efficacy. After the VSV infection for 12 h in AML12 cells, a low concentration of DCA/CDCA (40 μmol/L) could not inhibit the VSV infection in the CM condition. Interestingly, the antiviral efficiency of DCA/CDCA at 40 μmol/L concentration was evident under the BM condition (Fig. 2D). Furthermore, under BM condition, CDCA/DCA at 40 μmol/L concentration promoted the activation of interferon signaling, marked by the enhanced transcription of antiviral genes including *mIfnb1* and *mIsg56* in AML12 cells (Fig. 2E). We hypothesized that this was due to the differences in FXR activation in AML12 cells between CM and BM condition. Consistent with our hypothesis, CDCA/DCA increased *mNr0b2* transcription, a marker of FXR transcriptional activity, in AML12 cells cultured in CM, but had no significant influence on those cultured in BM (Fig. 2F). The FXR agonist GW4064 markedly suppressed poly (I:C)-induced transcription of *mIfnb1*, a marker of antiviral interferon signaling activation, indicating the inhibitory role of FXR in cellular antiviral response (Fig. 2G). Collectively, these findings suggest that FXR dampens the antiviral efficacy of BAs.

### 3.3 FXR inhibition decreases the virus infection in hepatic cells

**Figure 3:**
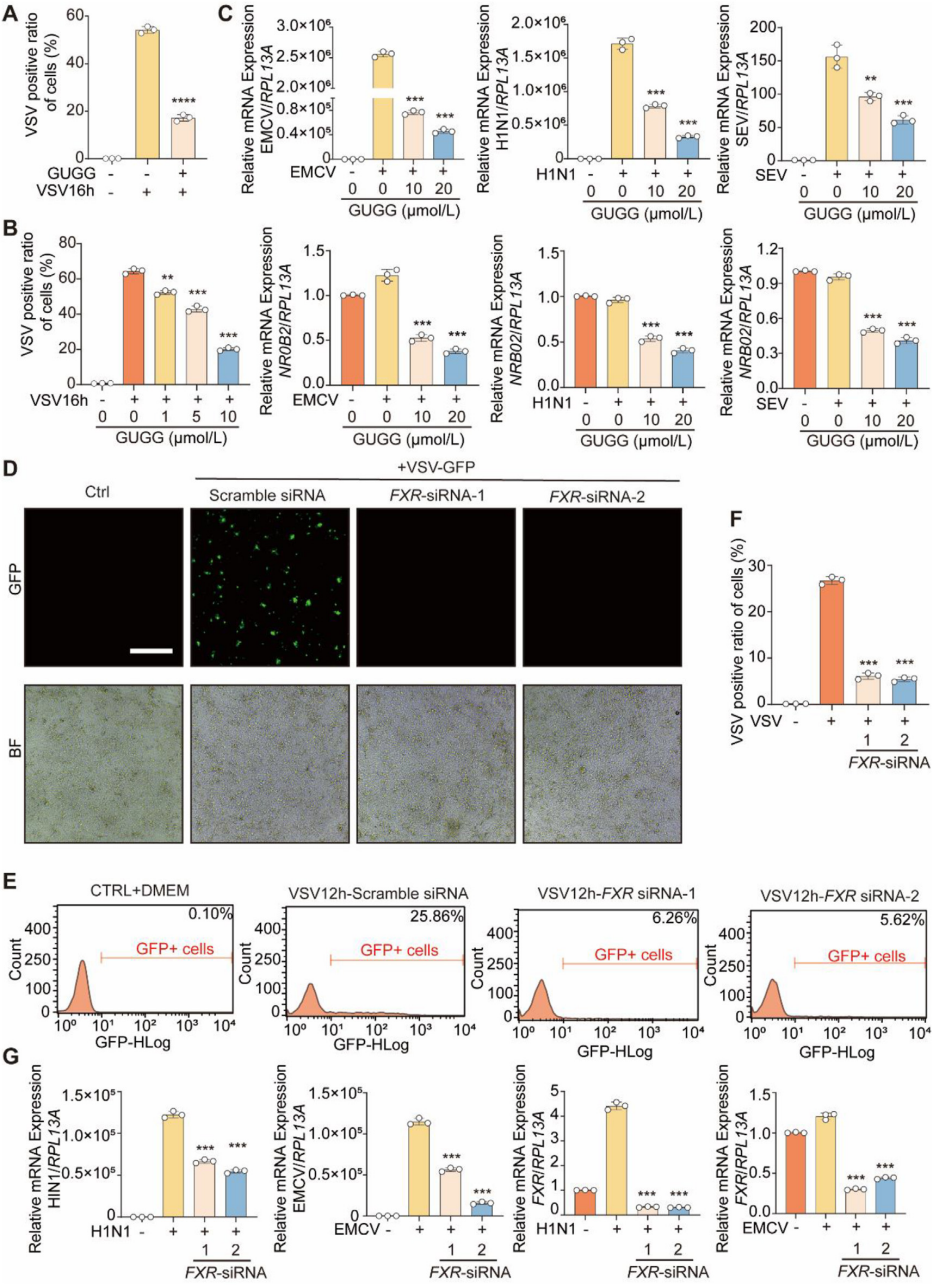
FXR inhibition decreases the virus infection in hepatic cells. (A) Flow cytometry analysis of VSV-GFP positive cells ratio in Huh7 cells infected with VSV-GFP for 16 h and treated with or without *Z*-Guggulsterone (GUGG) (10 μmol/L). (B) Flow cytometry analysis of VSV-GFP positive cells ratio in Huh7 cells infected with VSV-GFP for 16 h and treated with or without indicated concentration of GUGG. (C) qPCR analysis of EMCV, H1N1 and SEV or *NR0B2* mRNA expression in Huh7 cells infected with virus (EMCV/H1N1/SEV) for 12 h and treated with or without GUGG at indicated concentrations. (D) Immunofluorescence analysis of the amounts of VSV-GFP fluorescence of control or *FXR* siRNA-silencing Huh7 cells treated with or without VSV-GFP for 12 h. Scale bar: 100 μm. BF, Bright Field. (E, F). Flow cytometry analysis of VSV-GFP positive cells ratio of control or *FXR* siRNA-silencing Huh7 cells treated with or without VSV-GFP for 12 h. (G). qPCR analysis of H1N1/EMCV and *FXR* mRNA expression of control or *FXR* siRNA-silencing Huh7 cells treated with or without H1N1/EMCV for 12 h. (A–C) *n* = 3 per group; (F, G) *n* = 3 per group. The data are shown as mean ± SD and were analyzed by Student's *t*-test. ∗*P* < 0.05, ∗∗*P* < 0.01, ∗∗∗*P* < 0.001, ns = no significance.

To explore the potential role of FXR in impeding the efficiency of BAs in innate antiviral immunity, we assessed the antiviral efficacy of the FXR inhibitor *Z*-guggulsterone (GUGG). Our findings demonstrated that GUGG notably reduced the VSV-positive ratio in Huh7 cells (Fig. 3A), suggesting the FXR inhibition enhances the antiviral activity of the cells. In addition, GUGG blocked the VSV infection in a dose-dependent manner (Fig. 3B). Interestingly, GUGG did not exhibit antiviral activity under BM culture condition, which may be associated with the inactivation of FXR under BM condition (Supporting Information Fig. S1). In a dose-dependent manner, GUGG administration significantly inhibited viral infection and FXR activity in EMCV-, H1N1-, and SeV-infected Huh7 cells compared to the control cells (Fig. 3C). This suggests that FXR inhibition has broad-spectrum activity in suppressing infection by multiple RNA viruses. Next, to directly evaluate the effect of FXR on the antiviral response, we used a specific small interfering RNA (siRNA) to knockdown *FXR* transcription in Huh7 cells. The VSV-positive ratio was significantly decreased in *FXR*-silenced Huh7 cells compared to control cells (Fig. 3D). Flow cytometry also revealed that *FXR* knockdown reduced VSV infection in Huh7 cells (Fig. 3E and F). Similarly, viral replication, including that of EMCV and H1N1, was diminished in the *FXR*-silenced Huh7 cells (Fig. 3G). Overall, these findings reveal that targeting FXR may promote the antiviral response through an unknown mechanism.

### 3.4 FXR negatively regulates interferon signaling

**Figure 4:**
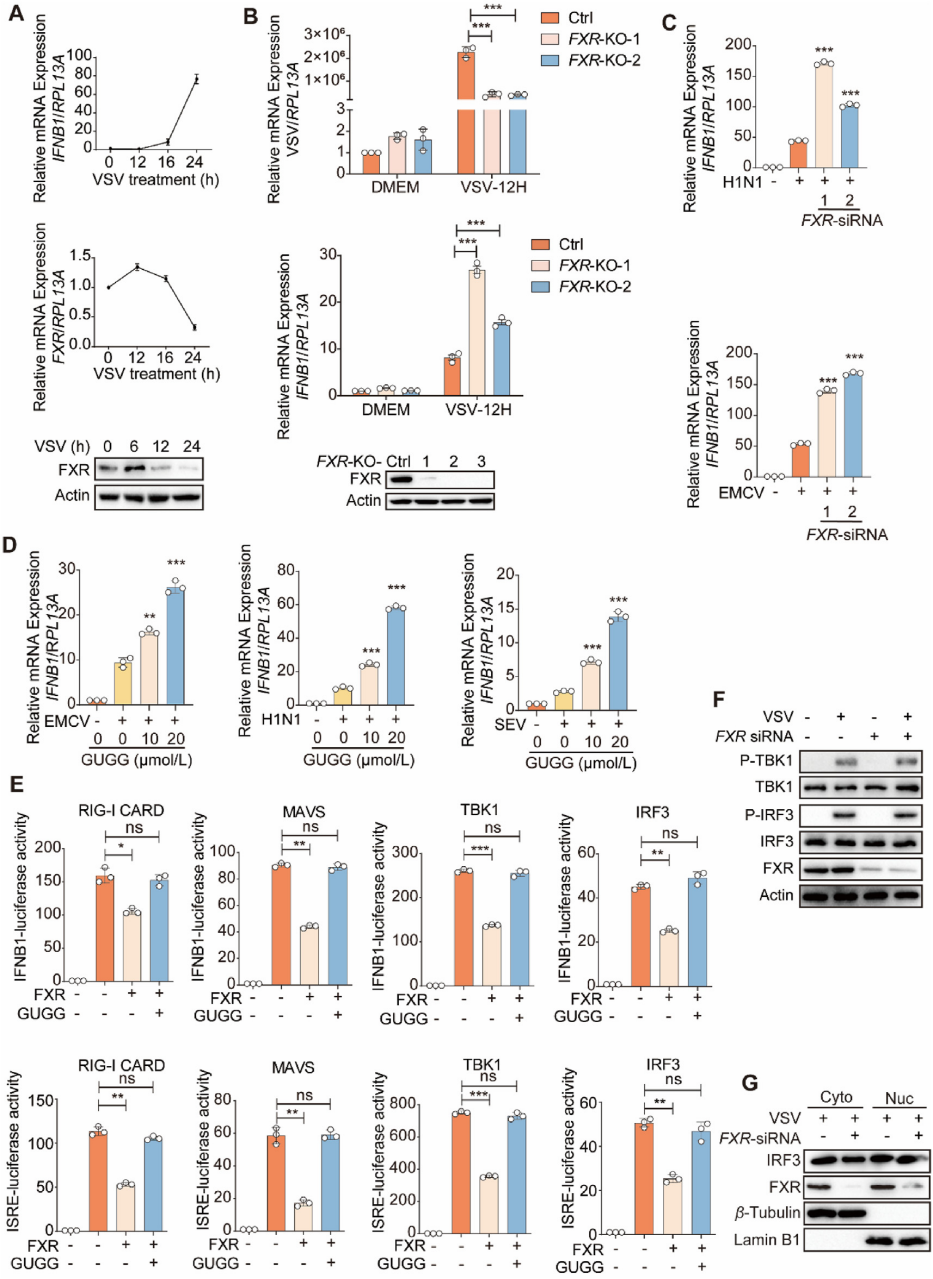
FXR negatively regulates the interferon signaling. (A) qPCR analysis of *IFNB1* and *FXR* mRNA expression in Huh7 cells treated with or without VSV for indicated times. FXR protein levels were analyzed by immunoblot analysis. (B) qPCR analysis of VSV and *IFNB1* mRNA expression of control or *FXR*-KO Huh7 cells treated with or without VSV for 12 h. FXR protein levels of control or *FXR*-KO Huh7 cells were analyzed by immunoblot analysis. (C) qPCR analysis of the *IFNB1* mRNA expression of control or *FXR* siRNA-silencing Huh7 cells treated with or without H1N1/EMCV for 12 h. (D) qPCR analysis of the *IFNB1* mRNA expression in Huh7 cells, and infected with EMCV/H1N1/SEV for 12 h or treated with or without GUGG (10/20 μmol/L). (E) Luciferase assays analysis of IFNB1/ISRE-dependent transcriptional activity in HEK293T cells transfected with RAD-I CARD, MAVS, TBK1 or IRF3. (F) Immunoblot analysis of P-TBK1 and P-IFR3 of control or *FXR* siRNA-silencing Huh7 cells infected with VSV for 12 h. (G) Immunoblot analysis of cytoplasm and nucleus IFR3 of control or *FXR* siRNA-silencing Huh7 cells infected with VSV for 12 h. (A–E) *n* = 3 per group. The data are shown as mean ± SD and were analyzed by Student's *t*-test. ∗*P* < 0.05, ∗∗*P* < 0.01, ∗∗∗*P* < 0.001, ns = no significance.

Interferon signaling is crucial for the antiviral response and is regarded as the primary target for viral evasion from the cellular innate immunity. Next, we examined whether the negative role of FXR in the antiviral response of cells was associated with changes in interferon signaling. Because GUGG enhanced the antiviral response in cells (Fig. 3C), we first investigated the transcription dynamics of *FXR* during the viral infection period of cells. *FXR* expression at the mRNA level was markedly increased in Huh7 cells in the early stage of VSV infection (<12 h) but decreased in the late stage of VSV infection (Fig. 4A). To assess the impact of FXR on the antiviral response and interferon signaling, we generated *FXR*-knockout Huh7 cell lines utilizing the CRISPR/Cas9 system. Compared to control cells, the replication of VSV was significantly decreased in *FXR*-deficient Huh7 cells. *FXR* deficiency strongly enhanced *IFNB1* expression in cells with VSV infection (Fig. 4B). Consistent with this observation, *FXR* knockdown also enhanced the mRNA expression of *IFNB1* in H1N1/EMCV-infected Huh7 cells (Fig. 4C). Additionally, our findings revealed that GUGG treatment significantly increased *IFNB1* production in Huh7 cells following infection with various RNA viruses, such as EMCV, H1N1, and SEV (Fig. 4D). Therefore, our results reveal that FXR may contribute to enhancement of viral replication by impairing interferon signaling.

In addition, we explored the detailed mechanism by which FXR inhibits the interferon signaling pathway under virus-infected condition. The activation of IFNB1-luciferase and interferon-stimulated response element (ISRE)-luciferase by overexpression of RIG-I CARD, MAVS, TBK1, and IRF3 was markedly suppressed by FXR overexpression but was rescued by GUGG treatment (Fig. 4E). The signaling cascade of interferon is derived from RIG-I, which transmits signals to MAVS through its CARD domain, subsequently activating MAVS–TBK1–IRF3. Given the inhibitory role of FXR on IRF3-initiated IFNB1-luciferase activity, it can be concluded that FXR inhibits interferon signaling by targeting IRF3. Additionally, immunoblotting results revealed that *FXR* knockdown did not impact the phosphorylation of TBK1 and IRF3 (Fig. 4F), indicating that FXR functions in interferon signaling at the later stage after IRF3 activation. *FXR* deficiency did not regulate the nuclear entry of IRF3 (Fig. 4G), indicating that FXR may regulate nuclear IRF3-mediated IFNB1 transcription. Therefore, our observations point to the certainty that FXR negatively regulates the activation of interferon signaling under RNA virus infection after the nuclear entry of IRF3.

### 3.5 FXR inhibits interferon signaling by blocking the transcriptional activity of IRF3

**Figure 5:**
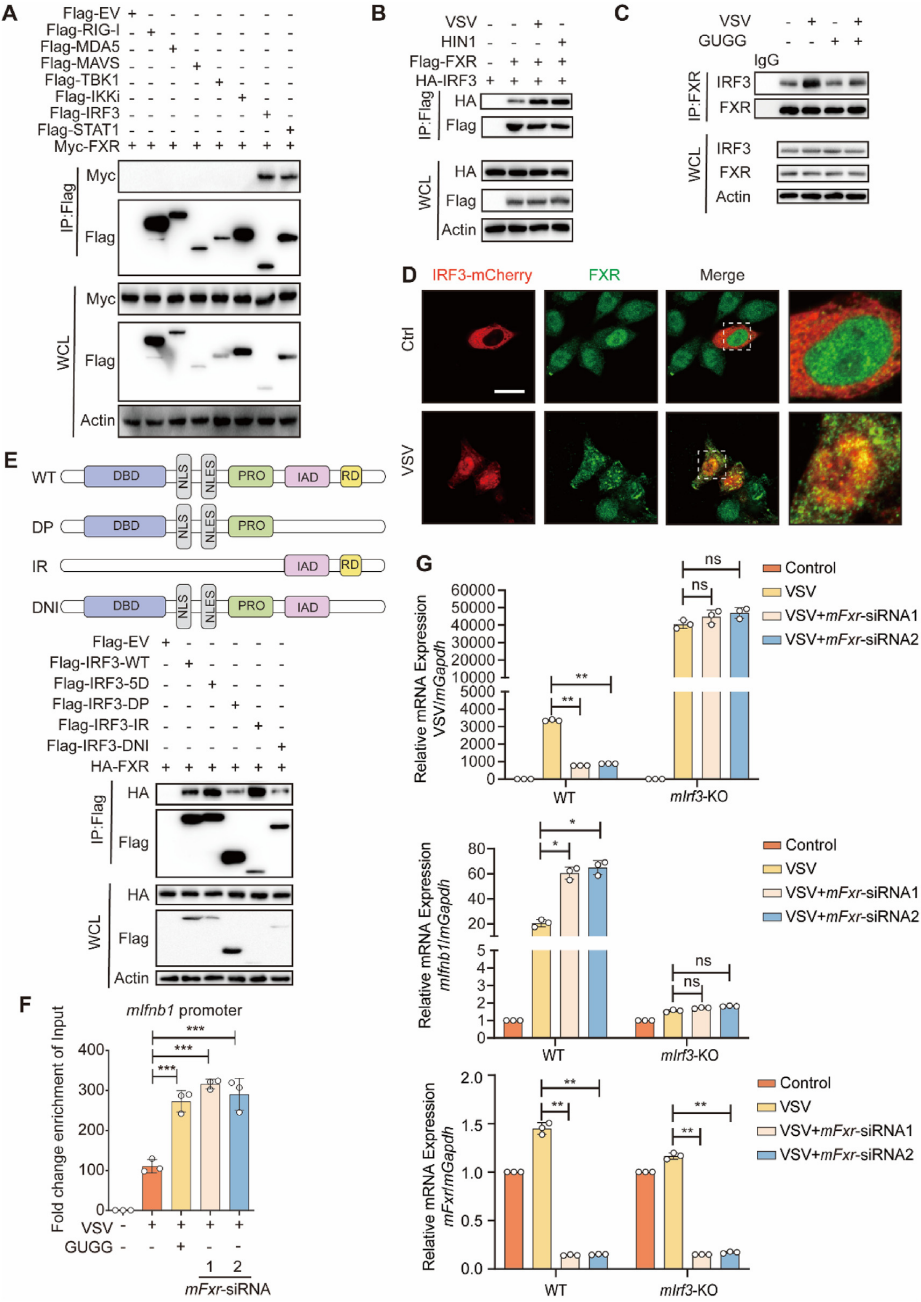
FXR inhibits interferon signaling by blocking the transcriptional activity of IRF3. (A) Coimmunoprecipitation and immunoassay analysis of extracts of HEK293T cells transfected with various combinations of expression vector for MYC-FXR and other indicated plasmids. (B) Coimmunoprecipitation and immunoassay analysis of extracts of Huh7 cells transfected with Flag-FXR, HA-IRF3, and treated with or without VSV/H1N1 for 12 h. (C) Huh7 cells were infected with or without VSV for 12 h following with the treatment of GUGG (10 μmol/L). FXR was immunoprecipitated from whole-cell lysates, and the protein levels of FXR and IRF3 in the precipitates were evaluated by immunoblotting. (D) Huh7 cells were transfected with IRF3-mCherry plasmid for 24 h, and then were treated with or without VSV for another 12 h. After fixation and FXR staining, the cells were subjected to confocal immunofluorescence analysis. Scale bar: 10 μm. (E) HEK293T cells were co-transfected with HA-FXR with Flag-IRF3 or its deletion mutants. The protein domain structures of the IRF3 deletions are depicted in a schematic diagram. Immunoprecipitation of Flag-tagged proteins was performed using anti-Flag beads, followed by verification through immunoblot analysis. (F) ChIP assays of the IRF3 binding to the promoter of *mIfnb1* gene under VSV infection following with GUGG treatment in control or *mFxr* siRNA-silencing AML12 cells. (G) qPCR analysis of the VSV and *mIfnb1* mRNA expression in control or *mIrf3*-KO AML12 cells following with or without *mFxr* knockdown under VSV infection for 12 h. (F, G) *n* = 3 per group. The data are shown as mean ± SD and were analyzed by Student's *t*-test. ∗*P* < 0.05, ∗∗*P* < 0.01, ∗∗∗*P* < 0.001, ns = no significance.

Given the negative regulatory role of FXR in interferon signaling, we delved into the molecular mechanism underlying FXR-mediating inhibition of interferon signaling. Interaction screening of FXR and the components of interferon signaling revealed a robust interaction between FXR and IRF3 or STAT1, key transcription factors in the interferon signaling pathway (Fig. 5A). Infection with VSV or H1N1 enhanced the interaction between FXR and IRF3 (Fig. 5B). In contrast, the administration of GUGG prevented the endogenous interaction of FXR and IRF3 during VSV infection (Fig. 5C), indicating that enhanced FXR interaction with IRF3 relies on FXR activation upon viral infection. Confocal imaging further depicted substantial colocalization of FXR with IRF3 puncta within the nucleus upon VSV infection (Fig. 5D). These results suggest that the interaction between FXR and IRF3 could be a crucial mechanism underlying FXR-mediated modulation of interferon signaling upon viral infection.

To explore the detailed interaction between FXR and IRF3, we constructed various IRF3 site mutants and domain-deletion mutants. IRF3-5D is the site mutant of mutating five phosphorylated serines of the regulated domain of IRF3 to aspartic acid to mimic the phosphorylated activation of IRF3. Our results revealed that phosphorylated IRF3 exhibited an increased interaction with FXR. We also found that the DP and DNI deletion mutants led to a reduced interaction of FXR with IRF3. This discovery highlights the necessity of the RD domain of IRF3 for FXR–IRF3 binding (Fig. 5E), indicating that the regulated domain of IRF3 plays a crucial role in the interaction between FXR and IRF3. These results demonstrate that FXR interacts with nuclear IRF3 and relies on the activation and phosphorylation of the regulated domain of IRF3, suggesting that FXR may directly influence the transcriptional activity of IRF3 in the nucleus. Thus, we next studied whether the genomic DNA binding of IRF3 is regulated by FXR. The results of the ChIP assays demonstrated that GUGG-mediated FXR inhibition or *FXR* deficiency enhanced IRF3 binding to the promoter of the interferon B1 gene, the target gene of IRF3, in VSV-infected cells (Fig. 5F). Moreover, *FXR* deficiency neither inhibited the replication of VSV nor enhanced the expression of *mIfnb1* in cells without IRF3 (Fig. 5G). Together, these results demonstrate that the FXR–IRF3 interaction impairs the transcriptional activity of IRF3 and damages the activation of interferon signaling and the antiviral response of cells.

### 3.6 FXR inhibition enhances the antiviral activity of CDCA

**Figure 6:**
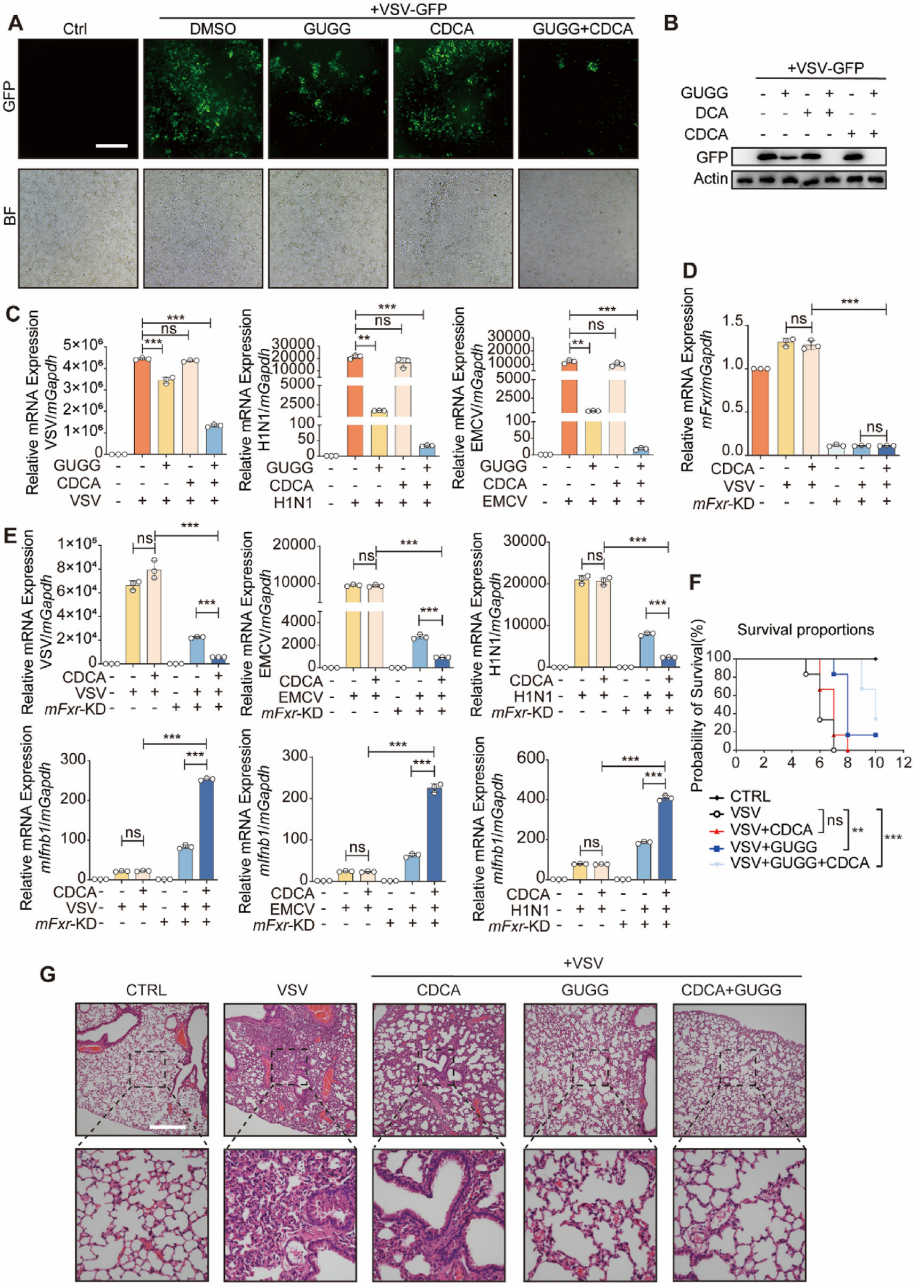
FXR inhibition enhances the antiviral activity of CDCA. (A) Immunofluorescence analysis of the amounts of VSV-GFP fluorescence in AML12 cells infected with or without VSV-GFP for 12 h following with or without treatment GUGG (10 μmol/L) or CDCA (40 μmol/L). BF, Bright Field. (B) Immunoblot analysis of the GFP level in AML12 cells infected with or without VSV-GFP for 12 h following with or without treatment GUGG (10 μmol/L), CDCA (40 μmol/L) or DCA (40 μmol/L). (C) qPCR analysis of the VSV/H1N1/EMCV mRNA expression in AML12 cells infected with or without VSV/H1N1/EMCV for 12 h following with or without treatment of GUGG (10 μmol/L) or CDCA (40 μmol/L). (D) qPCR analysis of the *mFxr* mRNA expression in control or *mFxr*-KD AML12 cells infected with or without VSV for 12 h following with or without treatment of CDCA (40 μmol/L). (E) qPCR analysis of the VSV/H1N1/EMCV mRNA expression and *mIfnb1* mRNA expression in control or *mFxr*-KD AML12 cells infected with or without VSV for 12 h following with or without treatment of CDCA (40 μmol/L) in CM condition. (F) Mouse survival was monitored (*n* = 6). (G) Hematoxylin and eosin staining of lung sections of mice. Scale bars = 100 μm. (C–E) *n* = 3 per group. The data are shown as mean ± SD and were analyzed by Student's *t*-test. (F) *n* = 6 per group. The data are analyzed by Kaplan–Meier methods. ∗*P* < 0.05, ∗∗*P* < 0.01, ∗∗∗*P* < 0.001, ns = no significance.

As the elimination of FXR hindered viral replication and enhanced interferon signaling, we proceeded to explore whether FXR played to the diminished efficiency of BAs in the antiviral response. Our investigation revealed that a low concentration of CDCA alone failed to inhibit VSV infection in cells. Compared to the application of GUGG or CDCA alone, the combination of GUGG with CDCA/DCA notably suppressed VSV infection in cells cultured in CM condition (Fig. 6A and B). GUGG treatment also enhanced the antiviral efficiency of BAs-related traditional medicines such as BBP (Supporting Information Fig. S2A and S2B). Moreover, combined treatment with GUGG and CDCA repressed the replication of several RNA viruses in cells cultured in CM condition, including VSV, H1N1, and EMCV (Fig. 6C). Additionally, *FXR* knockdown was performed to determine the role of *FXR* ablation in the antiviral activity of CDCA in cells cultured in CM condition (Fig. 6D). Our results revealed that *mFxr* deficiency enhanced the antiviral efficiency of CDCA upon infection with VSV, EMCV, or H1N1 and strongly promoted *mIfnb1* production simultaneously in cells cultured in CM condition (Fig. 6E). The measurement of IFNB1 protein levels revealed that treatment with GUGG in combination with CDCA significantly enhanced the protein expression and release of interferon (Fig. S2C). These findings indicate that the use of the FXR inhibitor GUGG in conjunction with CDCA can substantially promote interferon expression and the antiviral response of cells.

Additionally, to determine whether the synergy between FXR inactivation and CDCA treatment in promoting cellular interferon signaling is associated with TGR5, we assessed whether FXR inhibition by GUGG enhanced the CDCA-induced TGR5-mediated interferon signaling pathway. It is known that the CDCA–TGR5 axis promotes the interferon signaling pathway by enhancing the SRC-RIG-I interaction^12^. Our data showed that CDCA enhanced both TGR5–SRC and SRC–RIG-I interactions, indicating increased TGR5 activity. In contrast, FXR inhibition by GUGG did not affect these interactions (Supporting Information Fig. S3A and S3B). These results suggest that GUGG does not enhance the interferon signaling pathway *via* TGR5 activation, which reinforces by CDCA treatment. In conclusion, when cells are co-treated with CDCA and GUGG, GUGG specifically releases the inhibitory role of FXR on the IRF3 transcriptional activity and interferon expression without affecting the enhancing effect of CDCA on the TGR5-interferon axis (Fig. S3C).

Finally, we evaluated the antiviral efficiency of combined treatment with the FXR inhibitor GUGG and CDCA in VSV-infected mice. Our findings showed that GUGG-CDCA treatment significantly extended the final survival rate of mice infected with VSV (Fig. 6F). Consistent with these results, H&E staining of the lung tissue of mice revealed that the combined treatment with GUGG and CDCA reduced lung inflammatory lesions in VSV-infected mice (Fig. 6G), characterized by decreased infiltration of inflammatory cells into the lungs. It is noteworthy that VSV infection increased pathological alterations in the lung tissue. In contrast the combined treatment with CDCA and GUGG reduced such alterations in mice compared to the VSV-infected mice group (Supporting Information Fig. S4A). Combined treatment with CDCA and GUGG significantly reduced viral copy numbers in mouse lung tissue, concurrently enhancing the mRNA levels and protein expression of interferon (Fig. S4B and S4C). Together, these results demonstrate that FXR inhibition enhances the antiviral activity of CDCA and BA-related traditional medicines. Targeting FXR suppression may be a novel approach for accelerating the antiviral application of BAs.

## 4 Discussion

Emerging evidence suggests the importance of BAs metabolism and BAs receptors in the innate immune response^7^^,^^13^^,^^17^, ^18^, ^19^. One prominent discovery is that BAs stimulate antiviral innate immunity through the TGR5–*β*-arrestin–SRC pathway^12^. However, it is not completely understood whether other BAs receptors participate in the regulation of antiviral responses in cells. The key discovery of our study underscores the role of FXR, an important BAs receptor in hepatic cells, acts as a negative regulator of the cellular antiviral response by inhibiting the binding of IRF3 to the genome DNA and down-regulating interferon production. Additionally, our study demonstrated that GUGG, an FXR inhibitor, effectively inhibited viral replication and enhanced the expression of IFNB1 in virus-infected cells. These findings unveil the contradictory roles of the two BA receptors, shedding light on the intricate involvement of BAs and associated receptors in the cellular antiviral response. Furthermore, inhibition of FXR significantly enhanced the antiviral efficacy of CDCA in hepatic cells. While the antiviral activity of CDCA has been demonstrated, its clinical application in attenuating virus infection is limited to high concentrations (at least 100 μmol/L). Considering the safety and endogenous origin of BAs, CDCA, as a natural metabolite, can be utilized as a potential antiviral drug in combination with FXR inhibition.

CDCA, a prominent bile acid constituent, exerts its physiological effects through various receptors, including FXR and TGR5. TGR5, located on the cell membrane surface, detects both extracellular and intracellular bile acids, whereas FXR, an intracellular bile acid receptor, recognizes intracellular bile acids^1^. Previous studies have demonstrated that TGR5 enhances the activation of the interferon signaling pathway by increasing the phosphorylation levels of RIG-I, a key protein in the RNA sensing pathway. The activation of TGR5 by CDCA can consequently augment interferon expression and the antiviral response of cells^12^. However, it has been observed that the effective concentration of CDCA for antiviral activity is as high as 100 μmol/L^12^, whereas *in vitro* experiments indicate that the half-maximal effective concentration (EC_50_) of CDCA for TGR5 activation is approximately 15 μmol/L^20^. Within cells, the concentration of CDCA required to activate TGR5 and promote cell proliferation is only 5 μmol/L^21^. The notable contrast between the EC_50_ of CDCA for TGR5 activation and its effective antiviral concentration implies that there may be other factors influencing the antiviral effectiveness of CDCA in cells. Activation of TGR5 by BAs specifically regulates the tyrosine phosphorylation of antiviral signaling molecules, thereby enhancing interferon signaling and the cellular antiviral response^12^. While, previous studies have reported that the FXR inhibitor GUGG inhibits HCV replication^13^. Our study also found that the activation of FXR hampers the antiviral efficiency of BAs. TGR5 and FXR, the two main receptors of BAs^22^^,^^23^, may exert opposite functions in the innate antiviral immune response, as indicated by previous studies and our findings. Considering that both FXR and TGR5 within cells are the targeted proteins of CDCA, we hypothesize that FXR may limit the antiviral efficacy of CDCA.

The FXR protein contains two functional domains: the DNA-binding domain (DBD) and the ligand-binding and regulatory domain (RD). Previous studies have demonstrated that FXR utilizes the DBD to bind DNA and exert its transcriptional activity. Additionally, FXR can interact with other transcription factors through its RD domain, thereby suppressing gene transcription mediated by these factors^24^^,^^25^. We observed that knockdown or inhibition of FXR enhanced the cellular antiviral response (Figure 3, Figure 4). *FXR* knockdown did not affect the activation of the cytoplasmic interferon signaling pathway or nuclear translocation of IRF3 (Fig. 4F and G). Further exploration of the molecular mechanisms revealed a direct interaction between FXR and IRF3 (Fig. 5A–D). FXR predominantly interacted with IRF3 through its RD domain (Fig. 5E), indicating that FXR may impede the transcriptional activity of IRF3. Notably, *FXR* knockdown notably curtailed the DNA binding of IRF3 to the interferon promoter region (Fig. 5F). Lastly, in *mIrf3*-knockout cells, the regulation of interferon expression or the cellular antiviral response by *mFxr* knockdown was no longer evident (Fig. 5G). This suggests that FXR exerts its inhibitory effect on interferon expression and the cellular antiviral response by preventing the DNA binding of IRF3 to the promoter region of interferon. Treatment of cells with a combination of GUGG, an FXR inhibitor, and CDCA maintained the enhancing effect of CDCA on the TGR5–interferon signaling pathway while circumventing the inhibitory effect of CDCA-FXR on interferon transcription. Ultimately, this dual treatment resulted in the enhanced interferon expression and the inhibition of viral infection in cells.

FXR negatively regulates the innate immune response by inhibiting the activity of IRF3 and the production of interferon. Due to FXR's inhibitory influence on interferon signaling activation, the effectiveness of CDCA in combating viral infection within cells is suboptimal. These contradictory functions of TGR5 and FXR may contribute to the spatiotemporal regulation of interferon signaling. Research has demonstrated an increase in BAs synthesis within different cells upon virus infection, including immune and parenchymal cells, suggesting the potential involvement of BAs in cellular antiviral responses^12^. Our findings revealed higher expression of FXR in hepatocytes than in immune cells, indicating its potential function in hepatic cells. Overactivation of interferon signaling can lead to cell apoptosis and tissue damage^26^. Therefore, the negative effect and high expression of FXR in hepatic cells can promote cell survival during viral infection. Moreover, the positive contribution of TGR5 to interferon signaling activation ensures the transcription of antiviral proteins, including interferon and ISG56. The ‘yin and yang’ functions of the two BAs receptors maintain the balance between cell survival and viral inhibition during virus infection. However, how this balance is maintained deserves further detailed study.

Previous studies have offered insights into the mechanism of FXR inactivation in the BM condition. FXR loses its ability to inhibit transcription under cellular starvation^24^. FXR can interact with CREB, thereby inhibiting the CREB-mediated transcription of autophagy genes. FXR suppresses the transcription of autophagy-related genes under normal condition; however, this inhibition is relieved under cellular starvation condition, resulting in disruption of the FXR–CREB interaction^24^. We previously demonstrated that BM condition created for cells replicate a cellular starvation state^27^, which could result in the inactivation of FXR. Although our previous data showed that FXR was no longer activated under BM condition, further investigation of the molecular mechanisms underlying FXR inactivation under these conditions is currently ongoing. This evidence suggests that FXR loses its ability to bind to transcription-related factors under cellular starvation condition, thereby failing to exert transcriptional activation or inhibition.

Based on the aforementioned conclusions, we propose that activating TGR5 and inhibiting FXR may enhance the antiviral efficiency of BAs. Thus, FXR inhibitors are promising antiviral agents. Known FXR antagonists include DY268^28^, GUGG, UDCA, and G*β*MCA^29^, which suppress FXR activation through diverse mechanisms. GUGG alone did not affect FXR activity, but strongly inhibited FXR activation by CDCA^30^. GUGG interacts with the ligand-binding domain of FXR and disrupts the CDCA-induced interaction between FXR and its co-regulator^31^. However, UDCA cannot be involved in CDCA- or obeticholic acid (OCA)-stimulated FXR activation^32^, indicating that the FXR antagonistic function of UDCA may not enhance the antiviral activity of CDCA treatment in cells infected with RNA viruses. UDCA treatment alone prevented SARS-CoV-2 from invading cells by reducing the expression of ACE2, a cellular entry receptor for SARS-CoV-2. However, there is no evidence to suggest that UDCA inhibits SARS-CoV-2 by enhancing interferon signaling^14^. We found that UDCA treatment alone did not inhibit VSV replication or enhance interferon expression in cells (Fig. 1), which may be attributed to UDCA's inability to inhibit CDCA-stimulated FXR activation.

## 5 Conclusions

In summary, our study provides important molecular insights into how bile acid metabolism regulates innate antiviral responses in hepatic cells. Furthermore, our findings suggest the possibility of targeting FXR as an antiviral drug with high safety and efficacy. The combination of CDCA and FXR inhibitor treatment is a promising pharmaceutical strategy for the clinical control of RNA virus infection.

## Acknowledgments

This work was supported by the 10.13039/501100001809National Natural Science Foundation (NNSF) of China (Nos. 82371774 and 81901613), 10.13039/501100005090Beijing Nova Program (20230484342, China) and 10.13039/501100003453Natural Science Foundation of Guangdong Province (2020A1515011299, China).

## Author contributions

Xue Liang: Writing – original draft, Data curation, Methodology, Formal analysis, Investigation. Kunpeng Liu: Writing – original draft, Methodology, Investigation, Funding acquisition, Resources. Xin Jia: Data curation, Investigation. Cuiqin Cheng: Data curation, Formal analysis. Meiqi Zhang: Data curation, Investigation. Lingdong Kong: Methodology, Investigation. Qiqi Li: Investigation. Zhe Liu: Methodology. Min Li: Software. Junliang Li: Software. Yao Wang: Supervision, Writing – review & editing, Conceptualization, Funding acquisition, Resources, Project administration. Anlong Xu: Supervision, Validation, Writing – review & editing, Conceptualization, Funding acquisition, Project administration, Resources.

## Conflicts of interest

The authors declare no competing interests.

## Appendix A Supporting information

The following is the Supporting information to this article:
