# Supplementary material for: Suppressing FXR promotes antiviral effects of bile acids via enhancing the interferon transcription
Source: Acta Pharm Sin B. 2024 May 13;14(8):3513–27. doi: 10.1016/j.apsb.2024.05.005 (PMC11365379; doi:10.1016/j.apsb.2024.05.005)
Supplement: Multimedia component 1 [file mmc1.pdf]

Supporting Information for

Original article

Suppressing FXR promotes antiviral effects of bile acids *via* enhancing the interferon transcription

Xue Liang[a,†], Kunpeng Liu[b,†], Xin Jia[c,†], Cuiqin Cheng[a], Meiqi Zhang[a], Lingdong Kong[c], Qiqi Li[c], Zhe Liu[c], Min Li[a], Junliang Li[a], Yao Wang[a,d,*], Anlong Xu[a,d,*]

[a]*School of Life Sciences, Beijing University of Chinese Medicine, Beijing 100029, China*

[b]*Guangxi Key Laboratory of Special Biomedicine; School of Medicine, Guangxi University, Nanning, 530004, China*

[c]*Beijing Academy of Traditional Chinese Medicine, Beijing University of Chinese Medicine, Beijing 100029, China*

[d]*Beijing Research Institute of Chinese Medicine, Beijing University of Chinese Medicine, Beijing 100029, China*

Received 18 February 2024; received in revised form 9 April 2024; accepted 12 April 2024

*Corresponding authors.

E-mail addresses: xuanlong@bucm.edu.cn (Anlong Xu), yaowang@ bucm.edu.cn (Yao Wang).

[†]These authors contributed equally to this work

Supporting Information Figures S1–S4

Supporting Information Table S1

Figures S1

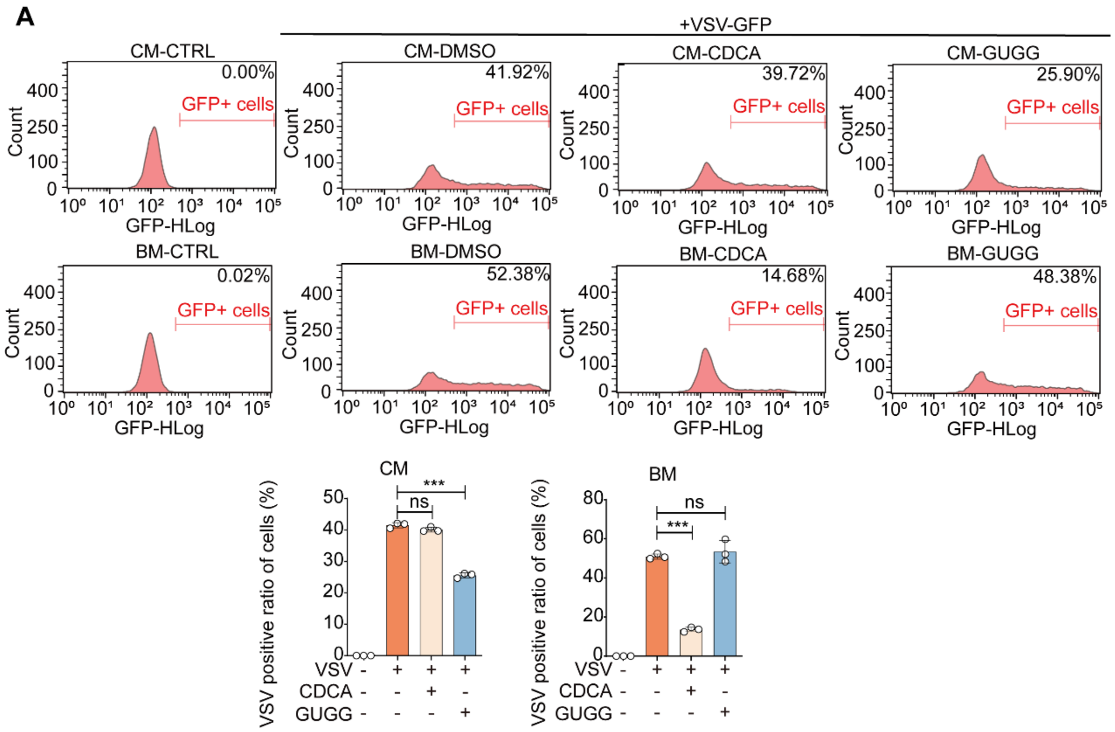

Figure S1 GUGG does not exhibit antiviral activity under BM culture conditions. Flow cytometry analysis of VSV-GFP positive cells ratio in AML12 cells cultured in CM/BM, and infected with or without VSV-GFP for 12 h following with or without the treatment of CDCA (40 μmol/L) or GUGG (10 μmol/L). *n* = 3 per group. The data are shown as mean ± SD and were analyzed by Student's *t*-test. *$P < 0.05$, **$P < 0.01$, ***$P < 0.001$, ns = no significance.

Figures S2

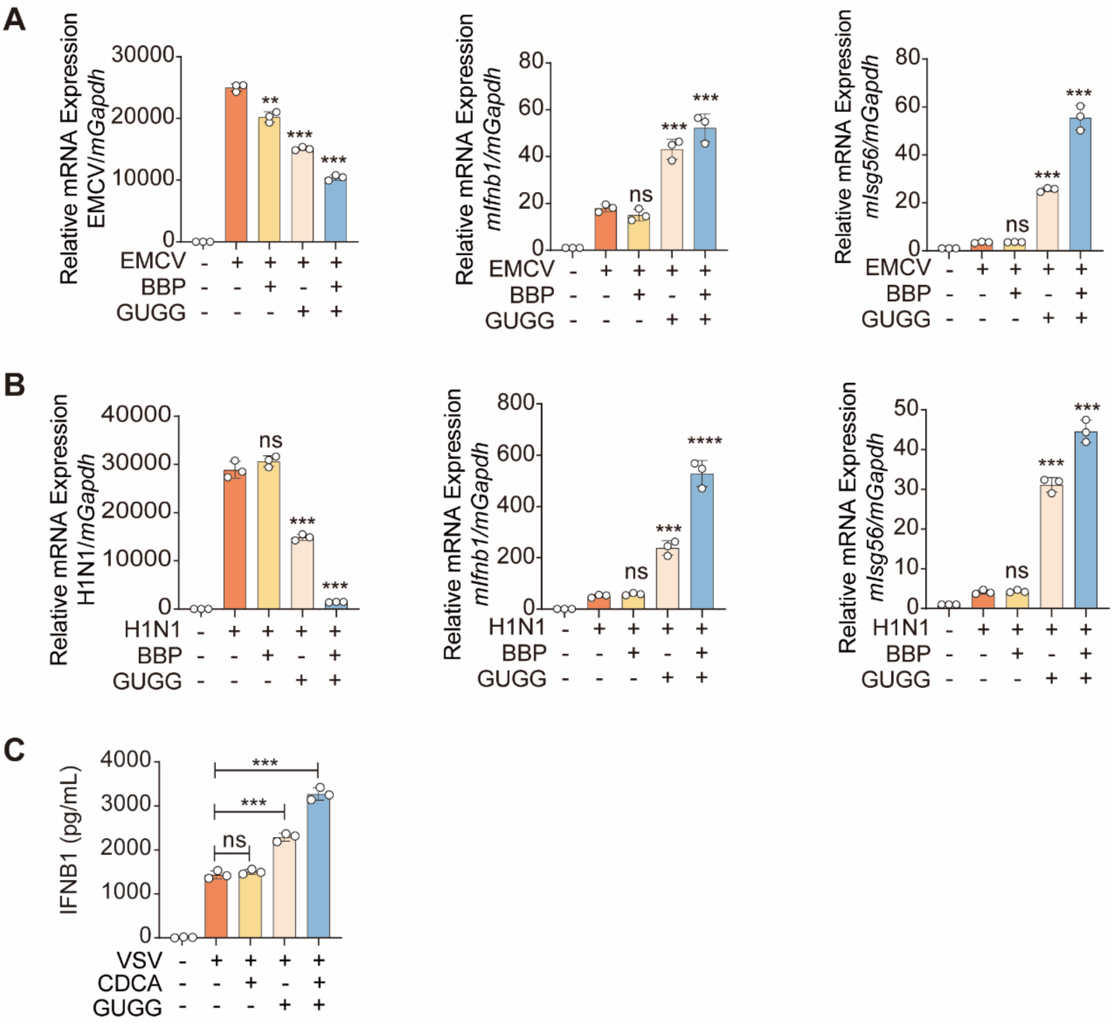

Figure S2 GUGG enhances the anti-viral efficiency of BAs-related traditional medicine (BBP) and CDCA. (A) qPCR analysis of the EMCV/*mIfnb1*/*mIsg56* mRNA expression in AML12 cells infected with or without EMCV for 12 h following with or without treatment of GUGG (10 µmol/L) or BBP (50 µmol/L). (B) qPCR analysis of the H1N1/*mIfnb1*/*mIsg56* mRNA expression in AML12 cells infected with or without H1N1 for 12 h following with or without treatment of GUGG (10 µmol/L) or BBP (50 µmol/L). (C) ELISA analysis of IFNB1 in AML12 cells infected with or without VSV-GFP for 12 h following with or without the treatment of CDCA (40 µmol/L) or GUGG (10 µmol/L). (A–C) n = 3 per group. The data are shown as mean ± SD and were analyzed by Student's t-test. *$P < 0.05$, **$P < 0.01$, ***$P < 0.001$, ns = no significance.

Figures S3

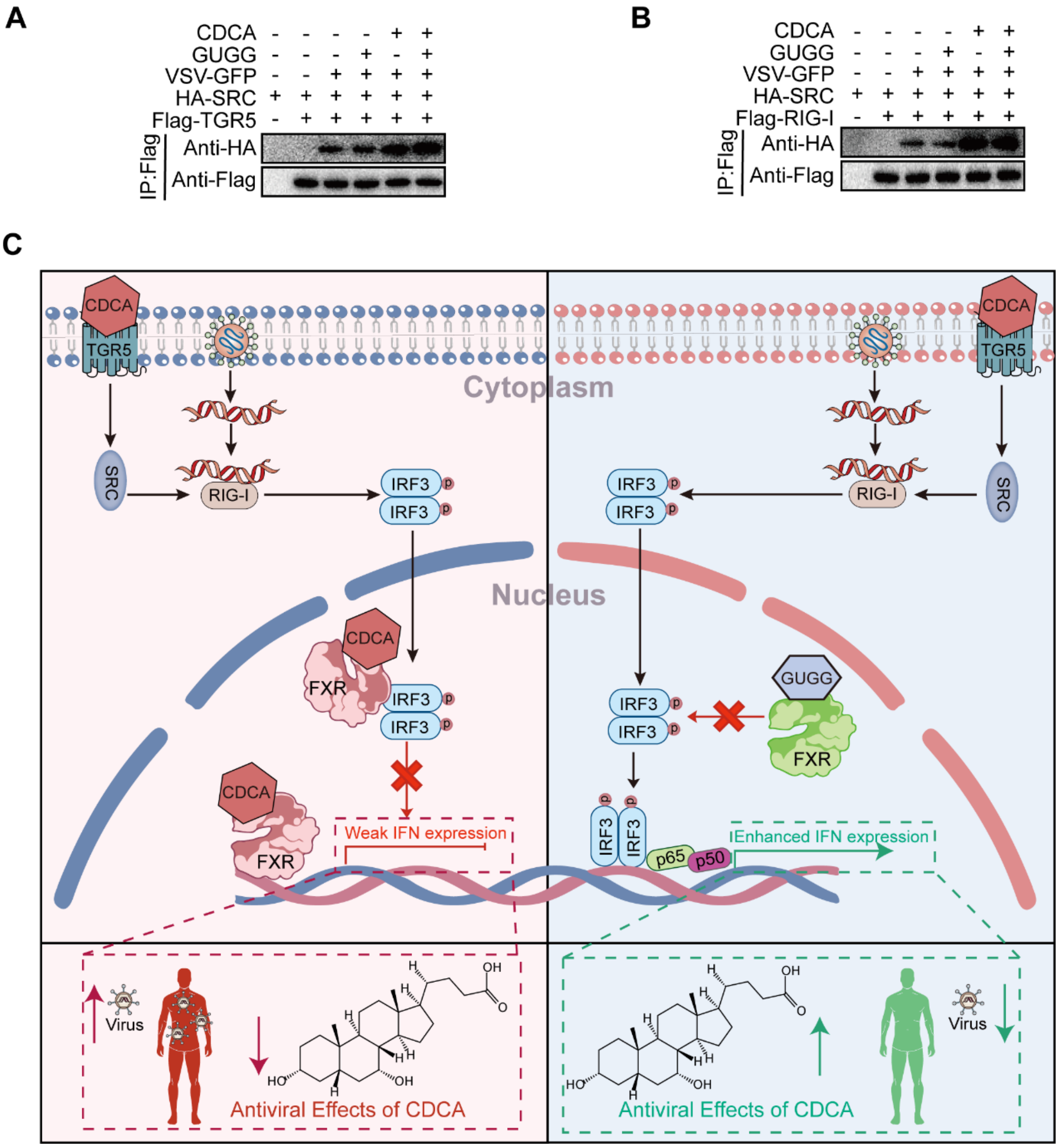

Figure S3 GUGG does not regulate both TGR5–SRC and SRC–RIG-I interactions enhanced by CDCA. (A) Coimmunoprecipitation and immunoassay analysis of extracts of Huh7 cells transfected with HA-SRC, Flag-TGR5, and infected with or without VSV-GFP for 12 h following with or without the treatment of CDCA (40 μmol/L) or GUGG (10 μmol/L). (B) Coimmunoprecipitation and immunoassay analysis of extracts of Huh7 cells transfected with HA-SRC, Flag-RIG-I, and infected with or without VSV-GFP for 12 h following with or without the treatment of CDCA (40 μmol/L) or GUGG (10 μmol/L). (C) Schematic representation of the FXR role in the antiviral effectiveness of CDCA.

Figures S4

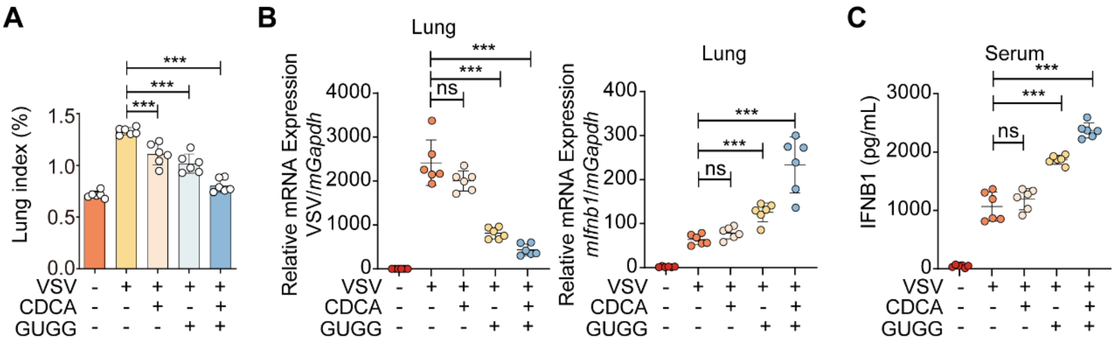

Figure S4 FXR inhibition by GUGG enhances the antiviral activity of CDCA. (A) Lung indexes were calculated in mice infected with or without VSV (2 × 10^8 pfu per mouse) following with or without the treatment of CDCA (10 mg/kg/day) or GUGG (10 mg/kg/day). (B) qPCR analysis of VSV mRNA expression in the lung of mice. (C) qPCR analysis of *mIfnb1* mRNA expression in the lung of mice. (D). ELISA analysis of IFNB1 in the serum of mice. (A–D) $n = 6$ per group. The data are shown as mean ± SD and were analyzed by Student's *t*-test. *$P < 0.05$, **$P < 0.01$, ***$P < 0.001$, ns = no significance.

Table S1 qPCR primers sequence.

Gene name	Forward primer (5′ to 3′)	Reverse primer (5′ to 3′)
VSV	ACGGCGTACTTCCAGATGG	CTCGGTTCAAGATCCAGGT
EMCV	TGGCGGGTGCAAGTTCAGTG	GGAGGAAGTGGGGGCTATGTTTAC
H1N1	CCCGGAAATAGCAGAAAGACCCAAAGTA	GCCGGACCCAAAGCCTCTACTCAGT
SeV	GCTGCCGACAAGGTGAGAGC	GCCCGCCATGCCTCTCTCTA
hIFNB1	GCTTGGATTCCTACAAAGAAGCA	ATAGATGGTCAATGCGGCGTC
hISG56	CCTCCTTGGGTTCGTCTACA	AGTGGCTGATATCTGGGTGC
hNR0B2	CCCCAAGGAATATGCCTGCC	TAGGGCGAAAGAAGAGGTCCC
hFXR	GACTTTGGACCATGAAGACCAG	GCCCAGACGGAAGTTTCTTATT
hRPL13A	GCCATCGTGGCTAAACAGGTA	GTTGGTGTTCATCCGCTTGC
mFxr	GCTTGATGTGCTACAAAAGCTG	CGTGGTGATGGTTGAATGTCC
mIfnb1	TCCTGCTGTGCTTCTCCACCACA	AAGTCCGCCCTGTAGGTGAGGTT
mIsg56	ACAGCAACCATGGGAGAGAATGCTG	ACGTAGGCCAGGAGGTTGTGCAT
mNr0b2	TGGGTCCCAAGGAGTATGC	GCTCCAAGACTTCACACAGTG
mGapdh	TGTGTCCGTCGTGGATCTGA	GCTTCACCACCTTCTTGAT
